# Pharmacokinetics of Phenprocoumon in Emergency Situations–Results of the Prospective Observational RADOA-Registry (Reversal Agent Use in Patients Treated with Direct Oral Anticoagulants or Vitamin K Antagonists Registry)

**DOI:** 10.3390/ph15111437

**Published:** 2022-11-19

**Authors:** Edelgard Lindhoff-Last, Ingvild Birschmann, Antonia J. Bidenharn, Joachim Kuhn, Simone Lindau, Stavros Konstantinides, Oliver Grottke, Ulrike Nowak-Göttl, Jessica Lucks, Barbara Zydek, Christian von Heymann, Ariane Sümnig, Jan Beyer-Westendorf, Sebastian Schellong, Patrick Meybohm, Andreas Greinacher, Eva Herrmann

**Affiliations:** 1 Coagulation Centre, Cardiology Angiology Centre Bethanien Hospital (CCB), 60389 Frankfurt, Germany; 2 Coagulation Research Centre, Cardiology Angiology Centre Bethanien Hospital (CCB), 60389 Frankfurt, Germany; 3 Institute for Laboratory and Transfusion Medicine, Heart and Diabetes Centre, Ruhr University, 44789 Bochum, Germany; 4 Institute of Biostatistics and Mathematical Modelling, Goethe University Frankfurt, 60590 Frankfurt, Germany; 5 Department of Anaesthesiology, Intensive Care Medicine and Pain Therapy, University Hospital Frankfurt, 60590 Frankfurt, Germany; 6 Center for Thrombosis and Hemostasis (CTH), University Medical Center, Johannes Gutenberg University, 55131 Mainz, Germany; 7 Department of Anaesthesiology, RWITH Aachen University Hospital, 52074 Aachen, Germany; 8 Institute of Clinical Chemistry, Thrombosis & Haemostasis Treatment Centre, University Hospital, 24105 Kiel-Lübeck, Germany; 9 Department of Anaesthesia, Intensive Care Medicine, Emergency Medicine and Pain Therapy, Vivantes Klinikum im Friedrichshain, 10249 Berlin, Germany; 10 Department of Immunology and Transfusion Medicine, Universitätsmedizin, 17475 Greifswald, Germany; 11 Department of Medicine 1, Division of Thrombosis & Hemostasis, Dresden University Clinic, 01307 Dresden, Germany; 12 Medical Department 2, Municipal Hospital, 01067 Dresden, Germany; 13 Department of Anaesthesiology, Intensive Care, Emergency and Pain Medicine, University Hospital Wuerzburg, 97080 Wuerzburg, Germany

**Keywords:** phenprocoumon, pharmacokinetics, emergency, major bleeding, urgent surgery, INR rebound

## Abstract

Background: Phenprocoumon has been used as an oral anticoagulant in patients with thromboembolic disease for more than 40 years. So far its pharmacokinetics have not been analyzed in emergency situations. Methods: Phenprocoumon-treated patients with major bleeding or urgent surgery were included in a prospective, observational registry. Phenprocoumon drug concentrations were analyzed in samples, collected as part of routine care using ultraperformance liquid chromatography tandem mass spectrometry. Moreover, anticoagulant intensity and drug half-life (t1/2) were calculated. Results: 115 patients were included. Phenprocoumon levels declined over time with a half-life of 5.27 and 5.29 days in patients with major bleedings (*n* = 82) and with urgent surgery (*n* = 33). Baseline phenprocoumon levels were 2.2 times higher in the bleeding group compared to the surgery group (1.92 vs. 0.87 ng/mL, *p* < 0.0001). International normalized ratio (INR) values decreased rapidly during the first 24 h. In 27.6% of patients a rebound of INR (recurrent increase > 1.5) was observed which was associated with significantly increased bleeding rates (22% vs. 4.2% in patients with or without INR rebound, *p* = 0.012). Conclusions: In emergency situations, the long half-life of phenprocoumon may cause INR rebound and associated recurrent bleedings. Optimal management may need to include repeated vitamin K supplementation over days.

## 1. Introduction

Patients with mechanical heart valves, nonvalvular atrial fibrillation, deep vein thrombosis or pulmonary embolism require oral anticoagulation [1,2,3,4]. Vitamin K antagonists (VKAs) have been used for these indications for more than 40 years. Anticoagulation quality is checked by regular measurements of the International Normalized Ratio (INR) [4,5]. While subtherapeutic anticoagulation (INR below target range) is associated with an elevated risk of ischemic events, supratherapeutic anticoagulation (INR above target range) increases the risk of bleeding [6]. To avoid this, the recommended therapeutic INR range is 2–3 for the most frequent indications [5]. The use of VKAs is complicated due to a narrow therapeutic window and an unpredictable dose-response relationship [7]. The most feared side effect of VKA treatment is major bleeding, including the life-threatening condition of intracerebral hemorrhage (ICH) [8]. In this situation, acute treatments aiming to reverse the coagulopathy caused by VKAs include the application of prothrombin complex concentrates (PCCs) [9] and vitamin K [8]. PCCs provide a rapid and effective method to replace deficient clotting factors and correct the INR, while vitamin K supplementation leads much more slowly to a normalization of the INR. Since vitamin K-dependent active clotting factors must first be carboxylated in the liver, INR correction is often observed only after 12 to 24 h, even if hepatic function is normal [10].

In Germany, phenprocoumon is the VKA which is almost exclusively used [11,12]. Compared to the VKA warfarin, the half-life of phenprocoumon is longer (72–270 h vs. 36–42 h for warfarin) [13]. So far, little is known about the pharmacokinetics of phenprocoumon in emergency situations such as major bleeding or urgent surgery.

We have therefore conducted the RADOA-registry (Reversal Agent use in patients treated with Direct Oral Anticoagulants or vitamin K antagonists). In this registry outcomes in consecutive patients treated with either direct oral anticoagulants (DOACs) or phenprocoumon and hospitalized for either major bleeding or urgent surgery were prospectively assessed [14,15,16,17]. In this paper, we analyze the baseline anticoagulant intensity on admission and the drug half-life (t1/2) of phenprocoumon in phenprocoumon-treated patients of the RADOA-registry [14].

## 2. Results

Overall, 131 patients in the RADOA registry were treated with phenprocoumon. One further patient received apixaban treatment in parallel to phenprocoumon treatment because of a medication error. This patient was excluded from the following analysis. In total, 851 plasma and/or serum samples were available for single or serial measurements of phenprocoumon concentrations from 115 patients (87.8%). These patients were included in the presented subgroup analysis.

Baseline characteristics of the patients are shown in Table 1. 71.3% (82/115) of patients were included because of life-threatening bleeding and 28.7% (33/115) were included because of emergency surgery. Median age was 78 years and the majority of patients was older than 75 years (70.8% of the bleeding patients and 51.5% of the patients with urgent surgery, *p* = 0.076). Median BMI was 26.3 kg/m^2^ without significant differences between the two groups (*p* = 0.251). Median creatinine clearance represented by Cockcroft–Gault formula was 59 and 68 mL/min, respectively (*p* = 0.651). Although patients with major bleeding presented slightly higher INR values at baseline, no significant differences between both groups were observed (*p* = 0.614).

Thirty day in-hospital mortality was 14.6% (12/82) and 12.1% (4/33) in the major bleeding and urgent surgery group (*p* = 1.0).

**Figure 1:**
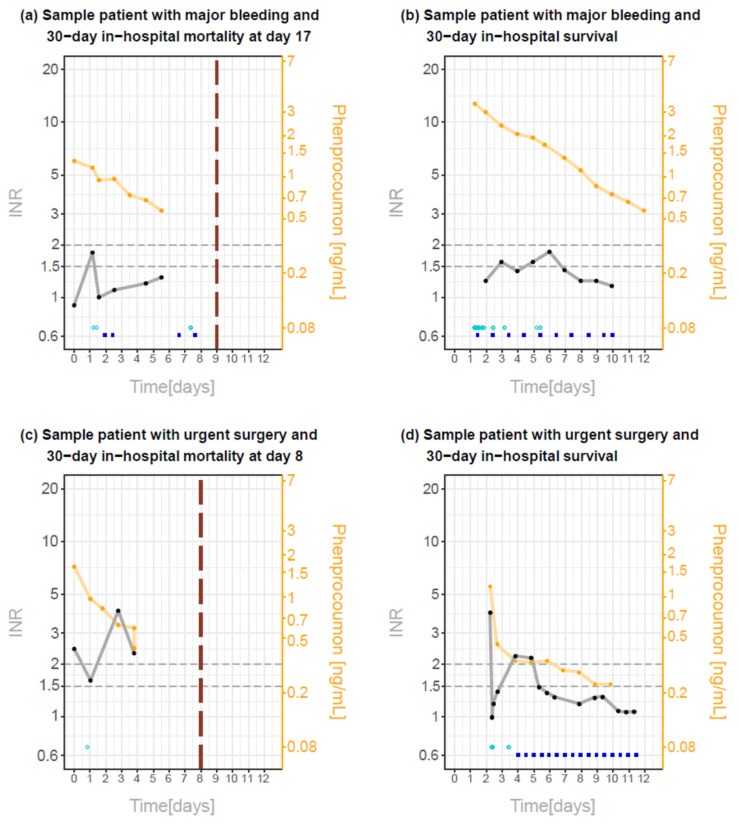
Pharmacokinetic phenprocoumon (orange dots and lines) and INR (black dots and lines) measurements over time in exemplary patients with major bleeding (Panel (**a**,**b**)) and with urgent surgery (Panel (**c**,**d**)). Patients in Panel (**a**,**c**) died during hospital stay, the vertical dashed line indicates the time of death. Blue filled circles (vitamin K) and cyan open circles (PCC) indicate the treatment time with vitamin K and PCC, respectively. Horizontal dashed lines mark INR levels of 1.5 and 2.0 as reference.

There was a considerable heterogeneity in the pharmacokinetics of phenprocoumon concentrations. Figure 1 shows exemplary patients with major bleeding with and without 30-day in-hospital mortality (Panel (a) and (b), respectively), as well as exemplary patients with urgent surgery with and without 30-day in-hospital mortality (Panel (c) and (d), respectively). 

**Figure 2:**
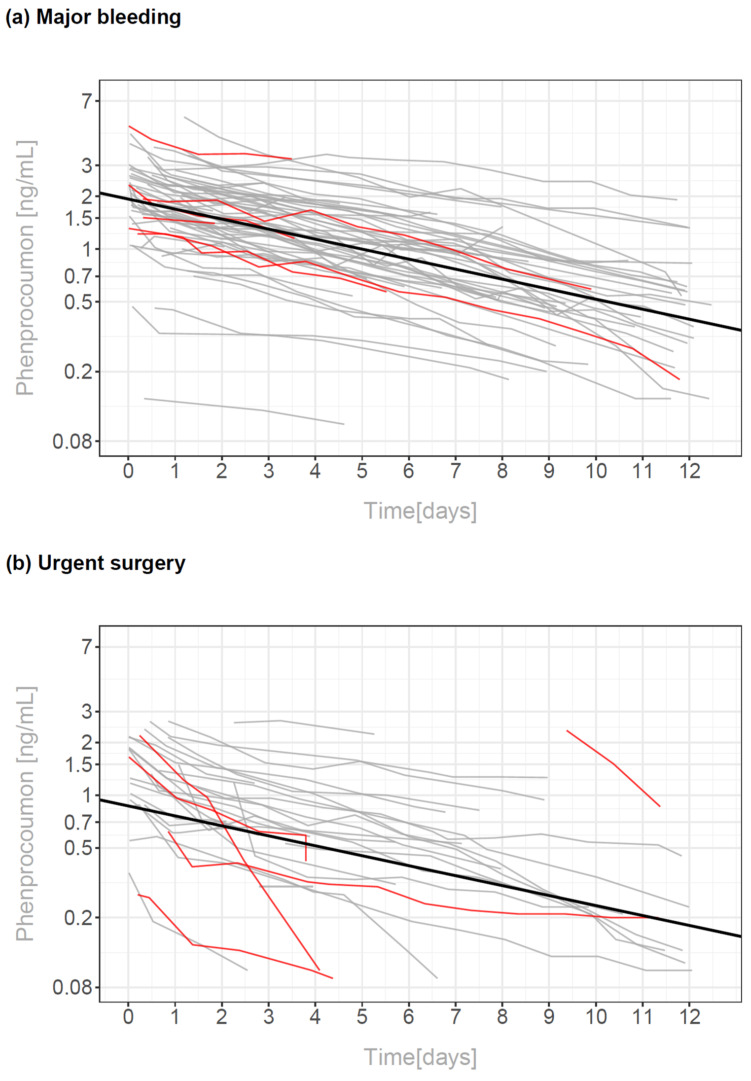
Course of phenprocoumon levels over time in patients with major bleedings (**a**) and in patients with urgent surgery (**b**). The regression lines were calculated by a single linear mixed model excluding data after early re-exposure to phenprocoumon and are shown as thick lines in each panel. Patients who died within 30 days after hospital admission are marked by red lines.

Pharmacokinetics were assessed with a mathematical linear mixed effect model. Phenprocoumon levels declined over time with a half-life of 5.27 and 5.29 days in patients with major bleedings and with urgent surgery (95% confidence interval: 4.59–6.36 and 4.25–7.01 days). The half-life estimations were not significantly different between the two groups. However, on admission fitted baseline levels were 2.2 times higher for the bleeding group compared to the surgery group (1.92 vs. 0.87 ng/mL, *p* < 0.0001). An overview for this fit is shown in Figure 2.

The majority of patients were treated with vitamin K (bleeding group: 82.9% 68/82, surgery group 63.6% 21/33, *p* = 0.0465) and/or PCC (bleeding group 69.5% 57/82, surgery group 78.8% 26/33, *p* = 0.365). Median cumulative numbers of vitamin K- and PCC-doses over time were 3.5 (interquartiles: 1–7) and 2 (interquartiles: 0–4).

INR levels typically decreased rapidly and 89.0% (97/109) of patients had at least one INR-level < 1.5 during the first 24 h of recorded measurements, while the INR-measurements of 11.0% (12/109) remained above 1.5. The remaining six patients had very few INR measurements which was mainly due to missing values because of a decision for palliative care treatment.

**Figure 3:**
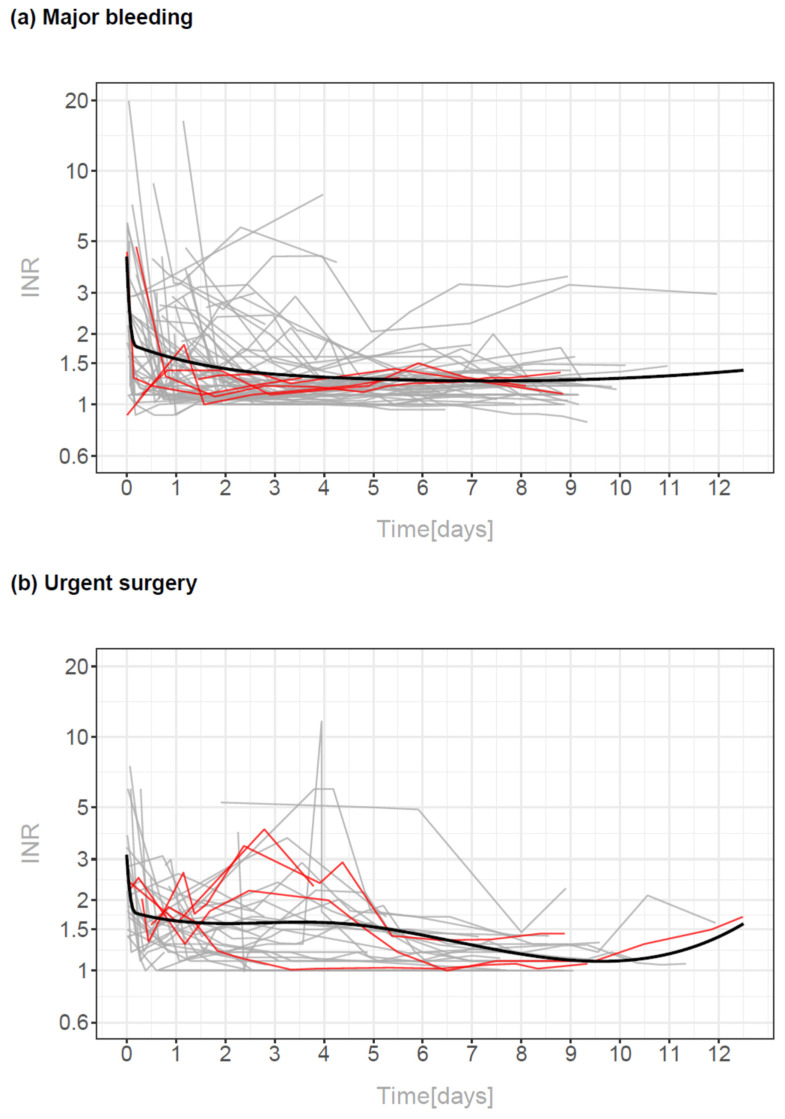
Course of INR levels in patients with major bleedings (**a**) and in patients with urgent surgery (**b**). The regression curves from a single linear mixed model with splines excluding data after early re-exposure to phenprocoumon are shown as thick lines in each panel. Individual INR-rebounds are documented. Patients who died within 30 days after hospital admission are marked by red lines.

Nevertheless, the INR-decrease slowed down early, leading to varying levels over time and sometimes even to increasing INR-levels later on (Figure 1 and Figure 3). More specifically, in 13 of 98 patients (13.3%) with an INR-level < 1.5 during the first 24 h, INR re-increased above 2 within the following 10 days and in additional 14 patients (14.3%) INR re-increased to levels above 1.5 within the following 10 days.

Recurrent bleedings were reported in 8.7% (10/115) of all patients and in 9.2% (9/98) of patients with an early INR decay < 1.5. In these 98 patients, recurrent bleedings were significantly associated with an INR rebound > 1.5, as 22% (6/27) of patients with an INR rebound had recurrent bleedings compared to 4.2% (3/71) of patients with an INR remaining below 1.5 (*p* = 0.012).

Multivariable logistic regression analysis including age > vs. ≤75 years, major bleeding vs. urgent surgery, occurrence of INR rebound and occurrence of recurrent bleedings as potential factors showed that only age above 75 years and recurrent bleedings were significantly and independently associated with increased 30-day-in-hospital mortality (*p* = 0.039 and *p* = 0.022).

Figure 3 shows the single measurements and the fitted curves for the INR-measurements over time differentiating between patients with major bleedings and patients with urgent surgery.

The deviation from a linear decay, as well as the difference between the groups in the dynamics around days 3 to 8, were significant (*p* < 0.001 and *p* = 0.001). Furthermore, the fitted INR- levels at baseline (on admission) did not differ significantly (*p* = 0.087) even though the fitted baseline levels in the bleeding group were numerically 1.3 times larger than those in the urgent surgery group.

## 3. Discussion

For decades, VKAs such as phenprocoumon have posed management challenges in emergency situations of anticoagulated patients [18,19,20,21,22,23]. So far, data on pharmacokinetics and pharmacodynamics of VKA in such settings are scarce. Our data shows that VKA-treated patients admitted to hospital for acute bleeding or emergency surgery often present with intense VKA activity and drug half-lifes of more than 5 days. As such, it is not surprising that, after immediate reversal management, more than 25% of patients develop INR-rebounds in the following days. Our study also demonstrates that repeat reversal strategies (repeat supplementation of vitamin K or PCC) are often required to counteract the rebound. Still, the 14% all-cause mortality within 30 days indicates the clinical importance of optimal VKA reversal strategies and the clinical impact of our analysis.

Phenprocoumon levels declined over time with a similar half-life of 5.27 and 5.29 days in patients with major bleedings and with urgent surgery, while the baseline phenprocoumon-levels were doubled in the bleeding patients compared to the surgical patients. In contrast, the baseline INR-levels were only 1.3 times higher in the bleeding group compared to the surgical group. This may be due to early PCC applications in the bleeding group to stop major hemorrhage which leads to a more rapid INR-decrease.

When immediate correction of the INR is required, clinicians frequently fail to administer vitamin K at the time coagulation factors (PCCs) are replaced. Failure to administer vitamin K may cause the INR to rise, usually 12 to 24 h after initial treatment, because the half-life of VKAs far exceeds the half-life of the coagulation factor complexes administered [24].

In 27.6% of VKA-treated patients included in the RADOA-registry, an INR rebound (INR > 1.5) occurred which was associated with recurrent bleeding in every 5th patient. Increases in INR 12–24 h after acute reversal therapy with PCCs have been reported in VKA-treated patients. In a retrospective study, 93 patients treated with warfarin received four factor-PCCs. 67.7% (63/93) were reversed for bleeding and 32.3% (30/93) for surgery [25]. Post-warfarin reversal, 71.4% (45/63) achieved INR correction at first draw, 87.3% (55/63) achieved INR correction within 24 h and 25.5% (14/55) experienced an INR rebound which is in agreement with our findings of the RADOA-registry. Of the 14 patients who experienced an INR rebound, 8 (57.1%) did not receive in parallel vitamin K [25].

The recent European Stroke Organisation Guideline consequently recommends the initial use of vitamin K (10 mg intravenously) in addition to fast reversal strategies including PCCs. This strategy is promoted to prevent INR rebounds in adult patients with ICH during warfarin treatment [8]. Due to the much longer half-life of phenprocoumon compared to warfarin, daily monitoring of the INR and repeated vitamin K administration may hence be useful and necessary for many days after admission to prevent INR rebound and recurrent bleeding.

We acknowledge that the RADOA registry has the typical limitations of a non-randomized observational registry. In daily clinical practice, registry data may be influenced by indication-related confounding. The small sample size is an additional limitation of the registry. On the other hand, randomized trials with patients in emergency situations and on oral anticoagulant treatments are difficult to perform. Additionally, it is ethically unacceptable to include placebo control groups in studies on life-threatening situations. Consecutive patients were included prospectively to obtain a high data quality and to minimize bias. Moreover, all enrolled patients were monitored on-site by an independent external supervisor. By enrolling consecutive patients, including those who could not provide informed consent, minimal selection bias can be expected [15].

On admission, phenprocoumon-treated patients experiencing emergency situations were on average 78 years old and the 30-day in-hospital mortality was 13.9%. These findings are in agreement with a recent retrospective Canadian cohort study including more than 2000 orally anticoagulated patients with major bleeding [26]. Even though the Canadian patients received warfarin instead of phenprocoumon, the mortality rate was comparable (15.2%) which supports the robustness of the RADOA-registry data.

In conclusion, the long half-life of phenprocoumon (5 days) and rebound INR over time associated with recurrent bleeding episodes particularly endanger elderly patients, who in themselves have a higher age-related risk of bleeding. Optimal management in emergency situations should not only include the immediate administration of PCCs but also concomitant long-term vitamin K administration. This treatment regimen may reduce complication rates. However, the results of our RADOA-registry analysis show that optimal management of VKA-treated patients in emergency situations is difficult to achieve, probably due to the complexity of clinical situations. Therefore, oral anticoagulation with phenprocoumon should be avoided in elderly patients and, wherever possible, a switch to DOACs with much shorter half-lives and reduced bleeding risks should be considered.

## 4. Materials and Methods

### 4.1. Study Design and Oversight

The prospective RADOA-registry is a German observational, noninterventional, open-label, investigator-initiated, multicenter registry which documents the management of severe bleeding and/or emergency surgery in patients treated with phenprocoumon or DOACs. The issues and purposes of the registry have been described previously [14]. Patients were followed prospectively up to 30 days after admitted to hospital. Participating centers were included only if they had 24 h interdisciplinary teams available to manage anticoagulant-related bleeding in specialized units (i.e., emergency departments and intensive care units). Approval of the study protocol was required by all relevant institutional review boards. External independent monitoring ensured 100% site source data validity.

### 4.2. Patients

The inclusion criteria of the RADOA registry were:

- Age > 18 years.
- Patients anticoagulated with DOACs or phenprocoumon with clinically overt major bleeding according to a modified definition according to the International Society of Thrombosis and Haemostasis for nonsurgical patients [27] that presented with at least one of the following criteria: symptomatic bleeding in a critical area or organ, such as intracranial, intraspinal, intraocular, retroperitoneal, intra-articular or pericardial, or intramuscular with compartment syndrome or acute life-threatening blood loss leading to hemodynamic instability and/or acute transfusion of two or more units of whole blood or red cells.
- Patients anticoagulated with DOACs or phenprocoumon needing an urgent surgical intervention within 24 h after admission.
- Patient inclusion lasted from 2014 to March 2018.

### 4.3. Ethics

Due to the emergency nature of the conditions under investigation, patient information and informed consent should not interfere with or delay acute treatment. With the approval of all ethics committees and institutional review boards, written informed consent was obtained from patients after the acute management phase. In the event of a patient’s inability to provide written informed consent, this was obtained from his/her legal representative. Data of patients who remained unconscious or died before a legal representative had been appointed were also included. This was explicitly approved by the ethical boards to prevent major bias caused by exclusion of the most severely affected patients [15]. The study complies with the Declaration of Helsinki.

**Clinical Trial Registration:** ClinicalTrials.gov Identifier: NCT01722786; URL: https://clinicaltrials.gov/ct2/show/NCT01722786?term=lindhoff-last&rank=9 accessed on 30 September 2022.

### 4.4. Substudy of the RADOA-Registry to Analyze Pharmacokinetics of Phenproumon

In a subgroup of patients included in the RADOA-registry phenprocoumon drug concentrations were analyzed in samples, collected as part of routine care using ultraperformance liquid chromatography tandem mass to analyze the pharmocokinetics (pks) of phenprocoumon [14]. Additional blood sampling for pks was not allowed which was due to the observational character of the registry. Therefore, time points of blood sampling were not prespecified and, thus, nonsystematic. Residual citrated plasma samples as well as serum samples were frozen and stored at the participating centers at –20 or –80 °C. They were later shipped on dry ice to the Institute for Laboratory and Transfusion Medicine, Heart and Diabetes Centre, Ruhr University Bochum, Bad Oeynhausen, Germany, where the ultraperformance liquid chromatography (UPLC)-MS/MS analysis of phenprocoumon concentrations was performed.

### 4.5. UPLC-MS/MS Measurement of Phenprocoumon

For measurement of phenprocoumon, a 2.1 × 50 mm reverse phase column (Waters, Acquity UPLC BEH C18, 1.7 µm) maintained at 60 °C was used for separation by an UPLC system directly coupled to a Waters TQ tandem mass spectrometer fitted with Z Spray ion source. An injection volume of 1.0 µL sample was used for each analysis. The flow rate of the mobile phase was set at 0.5 mL/min. The gradient program was 98%/2% water/methanol containing 0.1% formic acid for 0.2 min, followed by a linear gradient over 0.6 min of 95% methanol containing 0.1% formic acid. After the 95% methanol was maintained for 0.6 min, the mobile phase was revered to the initial state, and the run was terminated at 2 min. The mass spectrometer was operated in electrospray positive ionization mode. The system control and data acquisition were performed using MassLynx NT 4.1 software, with automated data processing by the MassLynx QuanLynx program provided with the instrument. Nitrogen was used as the nebulizing gas. Argon was used as the collision gas. Instrument settings were as follows: capillary voltage, 0.55 kV; source temperature, 110 °C; desolvation temperature, 460 °C; collision gas pressure, 3.2 × 10^−3^ mbar. Sample analysis was performed in the multiple reaction monitoring mode (MRM) of the mass spectrometer, with a dwell time of 0.025 s for all compounds and the mass transitions for phenprocoumon and its internal standard phenprocoumon-D5, m/z 281.2 > 203.1 and 286.1 > 203.1, respectively. Furthermore, we also monitored the slightly weaker *m/z* 281.2 > 174.9 ion of phenprocoumon and 286.1 > 174.9 ion of phenprocoumon-D5 for conformation.

### 4.6. Statistical Analysis

Quantitative variables are given as median and quartiles and compared between groups with the Wilcoxon-Mann-Whitney U test. Categorial data are described by absolute and relative frequencies and are compared with the exact Fisher test. Potential factors for 30 days in hospital mortality were analyzed with multivariable logistic regression analysis.

Pharmacokinetic and pharmacodynamic data were analyzed with linear mixed effect models and the significance of factors was estimated with a respective *t*-test. Quantifications after early re-exposure were excluded from the model evaluation. The half-life of phenprocoumon and 95% confidence intervals was then calculated from the regression coefficients of a pure linear mixed effect model for the log-phenprocoumon values with interactions between the decay and the groups (major bleeding vs. urgent surgery). The dynamics of INR did not follow a line but typically started with a fast decay which slows down early and may also differ from a linear decay afterwards. Therefore, the dynamics of INR was assessed with a B-spline model in a linear mixed effect model. Here, inner knots at 5 and 100 h after baseline were selected to account for a rapid early dynamic. Both linear mixed effect models use the Akaike information criterion (AIC) model selection criteria to select the final model.

All statistical tests of this explorative study were two-sided and use a significance level of alpha = 5% without correction for multiple tests. The statistical analysis was done with R (R Foundation for Statistical Computing, Vienna, Austria) using the “nlme” and “spline” packages.

## Figures and Tables

**Table 1:** Baseline characteristics of the patients included.

Variable	Total (*n* = 115)	Major Bleeding (*n* = 82)	Urgent Surgery (*n* = 33)
Male sex, *n* (%)	78 (67.8%)	53 (64.6%)	25 (75.8%)
Age (year) ^1^	78 (72–84)	79 (73–85)	76 (66–82)
BMI (kg/m^2^) ^1^	26.3 (23.5–29.2)	26.3 (23.9–29.6)	25.6 (22.4–29.2)
Dementia (%)	3 (2.6%)	1 (1.2%)	2 (6.1%)
Stroke/TIA (%)	26 (22.4%)	20 (24.4%)	6 (18.2%)
Cockcroft–Gault formula (mL/min) ^1^	66 (41–92)	59 (42–92)	68 (33–97)
INR ^1^	2.5 (2.8–3.7)	2.7 (1.6–4.0)	2.3 (1.9–2.9)
Known liver disease	5 (4.3%)	4 (4.9%)	1 (3.0%)
*Indication for anticoagulation*			
Non-valvular atrial fibrillation (%)	88 (76.5%)	65 (79.3%)	23 (69.7%)
CHADS-Vasc Score ^1^	5 (4–6)	5 (4–6)	5 (3.5–6.5)
HASBLED-Score ^1^	3 (2–3)	3 (2–3)	3 (2–3.25)
venous thromboembolism (%)	8 (7.0%)	7 (8.5%)	1 (3.0%)
Artificial heart valve (%)	8 (7.0%)	6 (7.3%)	2 (6.1%)
*Vascular risk factors*			
Hypertension (%)	92 (80%)	69 (84.1%)	23 (69.7%)
Diabetes mellitus (%)	35 (30.4%)	24 (29.3%)	11 (33.3%)
Hyperlipidemia (%)	43 (37.4%)	32 (39.0%)	11 (33.3%)
Smoking (%)	15 (13.0%)	10 (12.2%)	5 (15.2%)
*Type of bleeding* ^2^			
Intracranial/intraspinal, *n* (%)	-	53 (64.6%)	-
GI bleeding, *n* (%)	-	17 (20.7%)	-
Other locations, *n* (%)	-	13 (15.7%)	-
*Type of surgery* ^2^			
Trauma, *n* (%)	-	-	7 (21.2%)
Acute abdomen, *n* (%)	-	-	11 (33.3%)
Other surgery, *n* (%)	-	-	15 (45.5%)

^1^ median (1st–3rd quartile), ^2^ multiple types of bleeding locations and surgery are possible. BMI: body mass index, TIA: transient ischemic attack, INR: international normalized ratio, GI: gastrointestinal.

## Data Availability

Data is contained within the article.
